# Distribution of the Iberian *Calopteryx* Damselflies and Its Relation with Bioclimatic Belts: Evolutionary and Biogeographic Implications

**DOI:** 10.1673/031.010.6101

**Published:** 2010-06-11

**Authors:** David Outomuro, Antonio Torralba-Burrial, Francisco J. Ocharan

**Affiliations:** Departamento de Biologia de Organismos y Sistemas, Universidad de Oviedo, Oviedo, E-33071, Spain

**Keywords:** distribution maps, ecological requirements, relative frequencies, relative abundances, sexual selection, Iberian Peninsula

## Abstract

Using bioclimatic belts as habitat and distribution predictors, the present study examines the implications of the potential distributions of the three Iberian damselflies, *Calopteryx* Leach (Odonata: Calopterygidae), with the aim of investigating the possible consequences in specific interactions among the species from a sexual selection perspective and of discussing biogeographical patterns. To obtain the known distributions, the literature on this genus was reviewed, relating the resulting distributions to bioclimatic belts. Specific patterns related to bioclimatic belts were clearly observed in the Mediterranean region. The potential distribution maps and relative frequencies might involve latitudinal differences in relative abundances, *C. virgo meridionalis* Sélys being the most abundant species in the Eurosiberian region, *C. xanthostoma* (Charpentier) in the northern half of the Mediterranean region and *C. haemorrhoidalis* (Vander Linden) in the rest of this region. These differences might explain some previously described latitudinal differences in secondary sexual traits in the three species. Changes in relative abundances may modulate interactions among these species in terms of sexual selection and may produce sexual character displacement in this genus. *C. virgo meridionalis* distribution and ecological requirements explain its paleobiogeography as a species which took refuge in Iberia during the Würm glaciation. Finally, possible consequences in species distributions and interactions are discussed within a global climate change context.

## Introduction

A species distribution is determined by physical (e.g. temperature, rainfall patterns), biological (e.g. food availability, competition, predation) and historical factors. Establishing the importance of each factor is essential to generate accurate predictive models of distribution and to estimate expected changes in distribution and conservation status, given particular pressures (e.g. present climate change). In the case of organisms with complicated life cycles, each stage may respond to different factors or may show a response with a different intensity or threshold. Accordingly, the requirements of each stage must be considered.

Bioclimatic belts are the result of all physical variables affecting the landscape, since they are defined by thermal indexes, rainfall patterns and plant communities (Rivas-Martínez 1987). Moreover, these bioclimatic units correspond to an altitudinal zonation. Therefore, bioclimatic belts might predict suitable habitats for lotic species, since they involve major factors defining a river, such as altitude, slope, temperature and rainfall pattern, which will ultimately define current velocity, amount of water, substrate and level of dissolved oxygen.

*Calopteryx* Leach (Odonata: Calopterygidae) damselflies are excellent models to study the potential use of bioclimatic belts predicting distributions. They have a widespread distribution, their ecological requirements are well-known and, due to their conspicuousness, extensive data are available. Moreover, they display a high variability, showing a large number of subspecies or local forms (Askew 2004) and their phylogeny has been the object of several genetic studies (e.g. Dumont et al. 2005). Thus, their biogeography and variability may be discussed using present distributional ranges.

Furthermore, sexual selection processes have received a great deal of attention in the family Calopterygidae. Specific discrimination is based on the recognition of secondary sexual traits during a complex courtship ritual. In general, secondary traits would give information about the bearer's physical condition (Andersson 1994). In this family, some of the secondary sexual traits seem to be clearly dependent on the male condition (Grether 1996b) and are related to greater sexual fitness in different aspects (Grether 1996a, b; Rantala et al. 2000; Siva-Jothy 2000; Córdoba-Aguilar 2002; Contreras-Garduño et al. 2006, 2007). In fact, sexual selection processes in secondary sexual traits play a major role in specific divergence (Svensson et al. 2006). Intra- and interspecific sexual interactions are especially important in this family: male territorial contests among conspecifics (Grether 1996b; Córdoba-Aguilar 2002) or heterospecifics (Tynkkynen et al. 2004, 2005, 2006) and female mate choice during courtship (Siva-Jothy 1999). During these processes, secondary sexual characters are shown to the opponent or to the potential mate. Moreover, other selective forces are known, such as conspicuousness to predators (Grether 1997; Svensson and Friberg 2007) and prey (Grether and Grey 1996) and trade-off with immune response (Siva-Jothy 2000). Interspecific interactions (male-male competence and female mate choice) are especially relevant for our study, since they are modulated by the relative abundances of each species in sympatry (Tynkkynen et al. 2004, 2005, 2006). These sexual selection processes may lead to local events of secondary sexual traits displacement (Waage 1979; Tynkkynen et al. 2004, 2005, 2006) (see ‘evolutionary implications’).

The geography of the Iberian Peninsula creates a great diversity of lotic habitats, with differences between the Eurosiberian and Mediterranean regions, and, in the latter, between the areas of mountain, plateau or coast. As Iberian *Calopteryx* species have different ecological requirements (see ‘study species’), an associated distribution with respect to the bioclimatic belts may be expected, which will ultimately be reflected in the spatial frequency of each species. Together with species distribution, a higher relative frequency would involve a higher relative abundance of the species, which deeply influences interspecific interactions. Some forms and subspecies have been described on the Iberian Peninsula (see ‘study species’), which might be explained by interspecific sexual interactions.

The purpose of this study was to: 1) test the use of bioclimatic units in the preliminary prediction of distributions of Iberian *Calopteryx* species; 2) investigate whether differences in relative abundances exist on the Iberian Peninsula, in order to explain the variability of Iberian species from a sexual selection perspective; and 3) discuss paleobiogeography and future distributions within a global climate change context.

**Figure 1.**
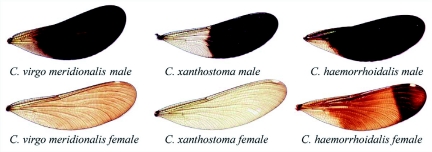
Images of Iberian *Calopteryx* wings. Males: *C. virgo meridionalis* has an extensively pigmented wing. In *C. xanthostoma*, pigmentation is only present from the wing apex to the node. *C. haemorrhoidalis* (subsp. *haemorrhoidalis*) shows a highly pigmented wing, although variation occurs between subspecies. Females: *C. virgo meridionalis* shows wing pigmentation over the entire wing. *C. xanthostoma* has no conspicuous pigmentation. *C. haemorrhoidalis* has a more pigmented apical spot on the hindwings. The relative position of the pseudopterostigma differs between species, being more apical in *C. xanthostoma*.

## Materials and Methods

### Study species

Three species of *Calopteryx* inhabit the Iberian-Balearic region: *Calopteryx virgo meridionalis* Sélys, 1873, *Calopteryx xanthostoma* (Charpentier, 1825) and *Calopteryx haemorrhoidalis* (Vander Linden, 1825). The distribution and habitat range of these species coincide partially and they frequently co-occur. Habitat selection seems to be mainly determined by larval requirements, defined principally by water temperature (Schütte and Schrimpf 2002), which strongly influences the global distribution of odonates (Corbet 1999). *C. haemorrhoidalis* appears in welloxygenated, clean and rather fast-flowing streams and rivers (Grand and Boudot 2006); *C. virgo meridionalis* occurs in cold, fast-flowing streams and rivers, with abundant waterside vegetation (Dijkstra and Lewington 2006; Grand and Boudot 2006); *C. xanthostoma* inhabits sunny, rather lowflowing rivers, with finer sediment and floating hydrophytes, even with pronounced drought periods (Goodyear 2000; Grand and Boudot 2006). *C. xanthostoma* larvae tolerate higher water temperatures and lower oxygen concentration than *C. virgo* larvae (Carchini and Rota 1985; Ferreras-Romero 1988).

Secondary sexual traits are conspicuous and plastic. In the Iberian species, males show a pigmented wing spot (Figure 1) and have the last three abdominal sternites specifically pigmented (reddish in *C. virgo meridionalis*, yellow in *C. xanthostoma* and carmine in *C. haemorrhoidalis*). Although all these traits are shown to the potential mate during courtship, apparently only wing pigmentation plays a role in specific discrimination (Svensson et al. 2007). Females are specifically distinguished by the relative position of the pseudopterostigma on the wing and wing pigmentation (Figure 1). Wing pigmentation is used for specific recognition and discrimination by males (Beukema 2004; Svensson et al. 2007). However, other species recognition cues might also be used by males, at least by *C. virgo* males (Svensson et al. 2007). The pseudopterostigma might also play a role in species discrimination (Outomuro D and Ocharan FJ, unpublished observations).

Some latitudinal differences with respect to secondary sexual traits have been recorded in Spain for the three species. From the northern slopes of the Cantabrian range to the Sistema Central range, males of *C. virgo meridionalis* and *C. xanthostoma* significantly have a proportionally more pigmented wing southwards. *C. virgo meridionalis* females have more pigmentation level southwards, while *C. xanthostoma* females have a shorter pseudopterostigma northwards (Ocharan Larrondo 1987; Outomuro D and Ocharan FJ, in prep.). Moreover, *C. haemorrhoidalis* have two subspecies on the Iberian Peninsula: *C. h. asturica* from the Cantabrian Eurosiberian region and *C. h. haemorrhoidalis* from the rest of the Peninsula (Ocharan 1983).

### Distribution maps

A bibliographic review of *Calopteryx* Iberian-Balearic records was carried out (see Appendix 1). Only records in which reliable 10 × 10 km UTM coordinates might be assigned were employed. References for *Calopteryx splendens* (Harris, 1782) and *Calopteryx virgo* (Linnaeus, 1758) (non-Iberian taxa) were considered as *C. xanthostoma* and *C. virgo meridionalis* records; *C. haemorrhoidalis* subspecies were not taken in account. Furthermore, our own unpublished records from several Spanish regions were included (principally from the Segura river basin; see Appendix 2). Obtained distribution maps must be understood as known (not real) species distribution, since many Iberian areas have scarce or no recorded data. Grid density in an area is not directly related to species frecuency, but to sampling effort; since this effort is equivalent for the three species, their relative values remain valid. Data were introduced in a geo-referenced matrix as 10 × 10 km UTM coordinates, obtaining species presence maps using ArcGis 9.1 (ESRI, Redlands, U.S.A.). A χ^2^ test was used to test whether the distribution of each species was random with respect to bioclimatic belts.

**Figure 2.**
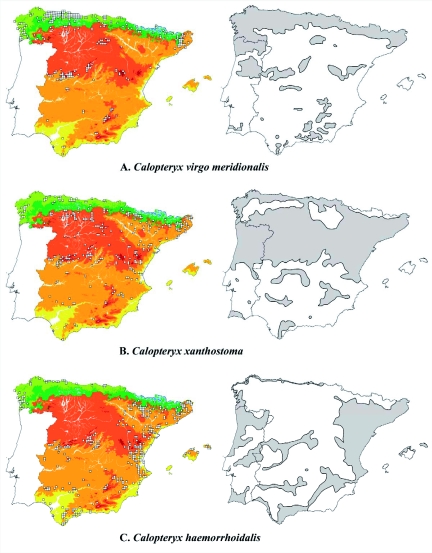
Distribution maps for the three Iberian species of *Calopteryx* damselflies. Left: Known distribution in Spain expressed as 10×10 km UTM grids on bioclimatic belts (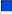 alpine, 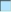 subalpine, 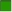 montane, 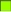 coline, 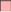 cryoromediterranean, 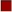 oromediterranean, 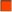 supramediterranean, 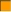 mesomediterranean, 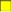 thermomediterranean). Right: Potential distribution on the Iberian Peninsula based on the association between species presence and bioclimatic belts. High quality figures are available online.

According to Rivas-Martínez (1987), two biogeographical regions may be recognised on the Iberian Peninsula: the Mediterranean and Eurosiberian regions, the boundary between which is located along the southern slopes of the Cantabrian and Pyrenean ranges and Galicia/northwest of Portugal. The above author recognises different bioclimatic belts (Figure 2) defined by thermal indexes. The Mediterranean region shows five belts on the Iberian Peninsula (from lower to higher altitude): thermo-, meso-, supra-, oro- and cryoromediterranean. The Eurosiberian region shows four belts: coline, montane, subalpine and alpine. Each belt is divided into horizons (the thermocoline horizon may also be considered a bioclimatic belt). Although bioclimatic belts are altitudinally zoned, altitude is not a variable used in their definition as bioclimatic units; therefore the altitude ranges are not similar or equivalent, especially between the two regions.

Distribution maps for Spain were superimposed on the map of bioclimatic belts, thus obtaining the UTM presence grid for each belt. Since more than one belt is possible inside each grid, there are more presence data for each belt than the total number of grids. Presence-corrected frequencies for each belt were calculated as the quotient between the total number of species presence grids in the belt and the total number of grids occupied by any of the three species. Finally, potential distribution maps are presented for each species on the Iberian Peninsula, including data from Portugal. These maps might indicate theoretical population fragmentation for each species. Due to the territorial behaviour of *Calopteryx* males (and homing behaviour in females), dispersion of these species is very low, although a small part of the population may disperse over relatively longer distances (e.g. more than 1 km, Stettmer 1996; Schütte et al. 1997). It may therefore be assumed that greater population fragmentation would also involve greater population isolation. However, these maps must be understood as potential maps, in which other biological, physical or chemical factors should not be forgotten, as predators, microclimatic effects, water quality or human impacts.

## Results and Discussion

### Species distribution

None of the species showed a random distribution related to bioclimatic belts (χ^2^ test for each species; d.f = 8; *P*<0.001). *C. virgo meridionalis* (Figure 2A and Table 1) is frequent in the Eurosiberian region (63.7% of Spanish records, despite the fact that this region only supposes 15% of Spanish territory), both in coline (52.1% of Eurosiberian records) and montane belts (41.2%)). It is very scarce in subalpine and alpine belts, since these only occur in high summits of the Cantabrian and Pyrenean ranges; its presence there might be associated with mountain valleys, where a low slope allows suitable waters for larval development. In the Mediterranean region, it is associated with major mountain ranges, generally appearing in the top horizon of the supramediterranean belt (50.5%), close to the oromediterranean belt. In coline, montane and supramediterranean belts, suitable conditions for larval development occur, i.e., cold rapidly-flowing rivers with abundant vegetation and rocky beds (Goodyear 2000; Dijkstra and Lewington 2006; Grand and Boudot 2006). In the Mediterranean region, only the presence of mountains permits these conditions. The southernmost Iberian populations occur in Los Alcornocales Natural Park (Cadiz), with meso- and thermomediterranean belts not associated with mountain ranges. This area has a Mediterranean climate with an Atlantic influence (high rainfall) (Carpintero et al. 2000) that allows suitable running waters for this species. The potential distribution map (Figure 2A) shows a typical oceanic species with a low frequency in the Mediterranean region. Three major groups of Iberian populations may be distinguished: northern, central (populations in the central mountain ranges) and southern groups. The northern group is continuously distributed over the Eurosiberian region, where the species shows a high relative frequency of presence (179 UTM grids, versus 83 for *C. xanthostoma* and 72 for *C. haemorrhoidalis*). Since the larval habitat is widespread, it may be assumed that higher relative frequency involves higher relative abundance of the species. Unfortunately, data are not available on population size to support this assumption. In the Mediterranean region, the relative frequency and abundance are lower (138 UTM grids, versus 247 for *C. xanthostoma* and 302 for *C. haemorrhoidalis*). In the central group, a certain degree of isolation may be observed among these populations and with respect to Eurosiberian group; both species frequency and abundance are intermediate. The southern group is widely fragmented and *C. virgo meridionalis* only appears in mountain streams supporting small populations; for that reason, both species frequency and relative abundance are lower compare to the central and northern groups.

**Table 1.**
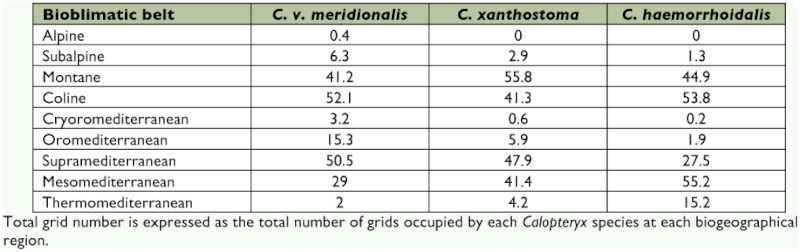
Percentage of grids occupied by a *Calopteryx* species in each bioclimatic belt.

*C. xanthostoma* (Figure 2B and Table 1) is principally distributed over the Mediterranean region (84.6% of Spanish records), where it is more frequent in the northern half; it is clearly associated with supra- (47.9% of Mediterranean records) and mesomediterranean (41.4%) belts. *C. xanthostoma* prefers less fast-flowing rivers (lower slope) than *C. virgo meridionalis.* Moreover, this species withstands lower oxygen levels and higher temperatures (Carchini and Rota 1985; Ferreras-Romero 1988). The supra- and mesomediterranean belts provide suitable conditions for these rivers. When it co-occurs with *C. virgo meridionalis*, its relative frequency in the mesomediterranean belt is higher. At higher altitudes (oromediterranean belt), the slope is too pronounced, while temperature may be too high and oxygen insufficient at lower altitudes (thermomediterranean belt). In the Cantabrian Eurosiberian region, *C. xanthostoma* is distributed in lower altitudes than *C. virgo meridionalis*; it is associated more with the coline belt. As ecological requirements are partially coincident between these two species (Carchini and Rota 1985; Ferreras-Romero 1988), *C. xanthostoma* co-occurs frequently with *C. virgo meridionalis* in this region. Population isolation (Figure 2B) is much lower than in *C. virgo meridionalis.* In the northern half of the Iberian Peninsula (Duero and Ebro basins), nearly continuous species distribution is observed, whereas populations appear to be more fragmented in the southern half. Isolation is not as clear as in *C. virgo meridionalis* since *C. xanthostoma* is less associated with mountain rivers. *C. xanthostoma* has a greater relative frequency and abundance compared to *C. virgo meridionalis* in the northern half of the Mediterranean region; *C. virgo meridionalis* is locally more frequent and abundant in mountain rivers. However, its relative frequency and abundance is much lower in the Eurosiberian region, especially in the Cantabrian area.

*C. haemorrhoidalis* is widely distributed over the east and south of the Iberian Peninsula, as well as the Ebro basin, the Sistema Central range and the coastal strip of Eurosiberian region (Figure 2C), presenting the typical distribution of a Mediterranean species. In the Mediterranean region, *C. haemorrhoidalis* (Table 1) is principally associated with meso- (55.2%) and supramediterranean (27.5%o) belts, and to a lesser extent with the thermomediterranean belt (15.2%). This is probably due to the fact that this species requires well-oxygenated streams (Grand and Boudot 2006). In most cases, therefore, oxygen will not be sufficient where temperature is high and slope is low (thermomediterranean belt). In the Eurosiberian region, it only appears in the montane (44.9%) and coline (53.8%) belts. However, presence in the montane belt only occurs in the Pyrenees, whereas on the Cantabrian Coast (and in the northwest of the Peninsula) it only inhabits the coline belt. In the Cantabrian coline belt, it is associated with coastal thermal enclaves, which may be called the thermocoline belt, characterised by warm winters and marked oceanity which imply little thermal amplitude between winter and summer (Rivas-Martínez 1987). *C. haemorrhoidalis* is totally absent in the Northern Sub-plateau (Duero basin). This is the only *Calopteryx* species which inhabits the Balearic Islands (Majorca and Minorca), associated with the mesomediterranean belt. There is a certain degree of population isolation (Figure 2C), distinguishing two major groups: 1) a Cantabrian group, consigned to the thermocoline belt; 2) the rest of the Iberian Peninsula (with more or less isolated populations). These are poorly connected by a narrow strip in the northwest of Spain. Relative frequency and abundance is much lower in the Eurosiberian region (restricted to the thermocoline belt), in relation to the other two species. The opposite situation occurs in the Mediterranean region.

**Figure 3.**
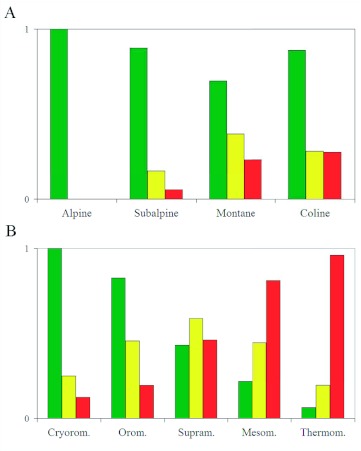
Corrected relative frequencies of Iberian *Calopteryx* species for each bioclimatic belt in Eurosiberian (A) and Mediterranean (B) regions. Species colour key: 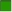 *C. virgo meridionalis*, 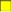 *C. xanthostoma*, 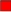 *C. haemorrhoidalis.* High quality figures are available online.

### Corrected relative frequencies

In the Eurosiberian region (Figure 3A), *C. virgo meridionalis* was the species with the highest relative frequency for the four belts. In contrast, *C. haemorrhoidalis* was the least frequent species (subalpine and montane belt data only refer to the Pyrenees). Species presence patterns related to bioclimatic belts and altitude may be observed. *C. xanthostoma* showed a maximum frequency in the montane belt, corresponding to medium river courses. *C. haemorrhoidalis* showed a maximum presence in the coline belt (in this case, the thermocoline belt), corresponding to low river courses. Results in the Mediterranean region (Figure 3B), were equivalent, though more significant; a clear frequency gradation being obtained. *C. virgo meridionalis* showed a decrease from a maximum in the cryoromediterranean belt to a minimum in the thermomediterranean belt. *C. haemorrhoidalis* presented the opposite results. These results are due to the fact that *C. virgo meridionalis* inhabits headwater stretches, while *C. haemorrhoidalis* appears in lower and/or more thermal stretches. Since *C. xanthostoma* inhabits medium river courses, it showed a maximum presence in the intermediate belt (supramediterranean), decreasing at higher and lower belts.

**Figure 4.**
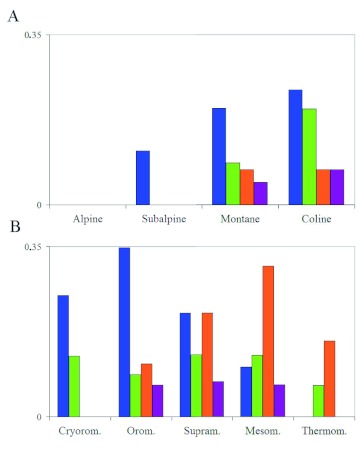
Corrected relative frequencies of Iberian *Calopteryx* species coexisting for each bioclimatic belt in Eurosiberian (A) and Mediterranean (B) regions. Associations colour key (*C. virgo meridionalis:* Cv; *C. xanthostoma*: Cx; *C. haemorrhoidalis:* Ch): 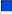 Cv+Cx, 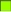 Cv+Ch, 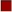 Cx+Ch, 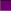 Cv+Cx+Ch. High quality figures are available online.

In the Eurosiberian region (Figure 4A), coexistence of the three species occurred in the coline belt (on the Cantabrian Coast) and the montane belt (in the Pyrenees). The most frequent species association was *C. virgo meridionalis* with *C. xanthostoma.* This may be easily explained by the fact that *C. haemorrhoidalis* generally inhabits thermal coastal rivers (Cantabrian coastal strip) or lower altitudes (Pyrenees) in this region. In the Mediterranean region (Figure 4B), the highest coexistence values for the three species occurred in meso-, supra- and oromediterranean belts. The most frequent association was *C. haemorrhoidalis* with *C. xanthostoma*, especially in the aforementioned belts. This is a logical finding, seeing as these two species present higher relative frequencies than *C. virgo meridionalis* in the Mediterranean region. The association between *C. virgo meridionalis* and *C. xanthostoma* occurred at higher belts.

### Evolutionary implications

The differences in relative abundances reported above may have a strong influence on interactions between species. As was mentioned above, sexual selection processes may involve secondary sexual characters displacement of isolated taxa in sympatry (e.g. Waage 1979) in such a way that these traits are modified divergently. This would be produced by specific recognition mistakes, since secondary characters are poorly-differentiated (De Marchi 1990). The displacement supposes an energy saving in reproductive effort (mating, sexual harassment and interspecific aggression) (Waage 1979; Mullen and Andrés 2007). Differences in relative abundance may create differential pressures on interspecific interactions that may in turn produce more or less noticeable secondary sexual character displacement depending on the abundance of the species that displaces the other (Tynkkynen et al. 2004, 2005, 2006). In Central Europe, *C. virgo virgo* males were more aggressive against the *C. splendens* males with larger wing spots, causing a displacement of this trait in the latter. Moreover, the degree of displacement depended on *C. virgo virgo* relative abundance. Wherever *C. virgo virgo* was more abundant, *C. splendens* had a smaller wing spot (Tynkkynen et al. 2004, 2005, 2006). This may be applied to females, though in terms of sexual harassment and interspecific matings. In fact, heterospecific matings are common, although reciprocal hybridization occurs at a low frequency (Tynkkynen et al. 2008).

Assuming the phylogenetic equivalence between Central European and Iberian species (Weekers et al. 2001), *C. virgo meridionalis* males might displace *C. xanthostoma* male secondary traits depending on their relative abundances. Female phenotypes would be ‘reinforced’ where species abundance is lower. In fact, where each species is less abundant, a new different form or subspecies with modified secondary traits appears. Morphological differences found by Ocharan Larrondo (1987) in Iberian *Calopteryx* populations may be due to a character displacement phenomenon. This would be produced in species populations with low relative abundance. The aforementioned author described *C. virgo meridionalis* females with a dark wing phenotype in the central Iberian Peninsula (Mediterranean region), where this species has a low relative abundance. Moreover, he described *C. xanthostoma* females with reduced or no pseudopterostigma on the northern slopes of the Cantabrian range (Eurosiberian region), where this species also presents a low relative abundance. Finally, Ocharan (1983) described *C. haemorrhoidalis asturica*, a subspecies consigned to Cantabrian Eurosiberian populations (thermocoline belt), where its relative abundance is also low. A recent study focussing on *C. virgo meridionalis* and *C. xanthostoma* showed these differences once again in Iberian populations, from the northern slopes of the Cantabrian range to the central Iberian Peninsula (D. Outomuro D and Ocharan FJ, in prep.). In fact, coloration differences were found in secondary traits not only in females, but also in wing spot extension in males, showing an increase of pigmentation southwards. Differences show a clinal variation supported by clinal relative abundance. Furthermore, other sexual character differences were found in areas where the three Iberian *Calopteryx* species coexist, suggesting a possible role of *C. haemorrhoidalis* in character displacement on *C. virgo meridionalis* (Outomuro D and Ocharan FJ, unpublished observations). These described variations may not be clearly explained by environmental factors (e.g. altitude) or other hypotheses for melanism such as thermoregulation, cryptic coloration, protection from ultraviolet radiations, disease resistance, etc. (Outomuro D and Ocharan FJ, unpublished observations). However, further studies are necessary to explain these forms or subspecies inhabiting the Iberian Peninsula, especially genetic studies between Iberian populations, since recent works are insufficient and too general (e. g. Weekers et al. 2001).

### Biogeography and implications in a climate change context

During the last major glaciation (Würm glaciation, Pleistocene), the western Mediterranean would have been one of the refugia for the genus *Calopteryx.* After this period, *Calopteryx* taxa would have reinvaded western Europe from the western Mediterranean refugium and centralwestern Asia refugium/refugia (Weekers et al. 2001). *C. virgo meridionalis* described distribution and other facts support the hypothesis that *C. virgo meridionalis* also stayed in the western Mediterranean refugium during the Pleistocene (likewise *C. xanthostoma* and *C. haemorrhoidalis*: Dumont et al. 2005): 1) existence of relict southern populations (also in Morocco, see below), corresponding to the Iberia refugium, and 2) excluding distributions of *C. virgo meridionalis* and *C. virgo virgo* and presence of intermediate forms in sympatry (Maibach 1986). The separation of these two subspecies from an ancient one might have been due to isolation during the last major glaciation.

*C. virgo meridionalis* shows typically relict populations. In the southernmost regions of the Iberian Peninsula, it only persists in microclimatic refugia; for instance, spots in the south and at high altitudes in the southern mountain ranges. Moreover, in Africa, only two relict locations are known in Morocco (Riff mountains over 1000 m: Jacquemin and Boudot 1999); two old records (Sélys 1871; Martin 1910) in northern Algeria have not been reconfirmed (Samraoui and Menaï 1999). Recent dispersion from southern Europe to northern Africa is unlikely, since southern Iberian populations sustain a low number of individuals (Ferreras-Romero M, University of Pablo Olavide, Seville, Spain, personal communication).

Mediterranean Peninsulas might have acted as glacial refugia during Würm glaciation. Later dispersion might have involved a clash with congeneric species in Central Europe. That is the case of *Calopteryx* Iberian taxa (though not with *C. haemorrhoidalis*). Many species distributions are subdivided by narrow hybrid zones which would have been produced by the clash between two divergent genomes, both expanding their distributional range from glacial refugia. One such hybrid zone is located in central-southern France (Hewitt 2000). An introgression zone between *C. xanthostoma* and *C. splendens* based on morphological characters has been described in this hybrid zone (Dumont et al. 1993), and another may possibly exist between *C. virgo meridionalis* and *C. virgo virgo*, since their distribution is continuous from Central Europe to the Iberian Peninsula. Maibach (1986) described intermediate forms between these two subspecies in Central France, where the contact zone is supposedly located. Unfortunately, to our knowledge, no more information on introgression zones between *C. virgo* subspecies has been reported in France. At least another *C. virgo* subspecies has been described, named as *Calopteryx virgo festiva* (Brullé, 1832), which inhabits the southern Balkans and Turkey (Dijkstra and Lewington 2006). The Balkans also acted as a glacial refugium and a post-glacial source of species for eastern and western areas (Hewitt 2000). *C. virgo meridionalis* and *C. virgo festiva* might have been dispersed from their refugia (Iberia and the Balkans) to Europe after the Würm glaciation and would have clashed against the nominal subspecies *C virgo virgo* originating from Asian réfugia. Several *C. splendens* subspecies have likewise been reported, most of which come from southern Mediterranean peninsulas, forming introgression strips with *C. splendens splendens* (Grand and Boudot 2006).

It is believed that many species will change their distributional range to higher altitudes and/or latitudes as a response of climate warming. Headwater streams are also sensitive to climate change and some scarce macroinvertebrate taxa might run the risk of local extinction due to an increase in winter temperatures (Durance and Ormerod 2007). Expansion northwards of the distributional range of 34 non-migratory Odonata species was documented in Great Britain between 1960 and 1995, apparently as a result of climate change (Hickling et al. 2005). Faunistic references are increasingly more frequent nowadays, supporting northern expansion of some Odonata species, as well as an increase in migratory flows to the Britain Isles. However, a possible increase in sampling efforts should be taken into account; so new data for a species do not necessarily mean that it did not previously exist in those areas (Askew 2004). Distributional range expansions to higher latitudes or/and latitudes in the northern hemisphere have not only been documented in dragonflies, but also in butterflies, birds, lichens, alpine flora, forests and even in a lagomorph species (for a review, see Parmesan 2006).

A general increase in temperature and decrease in rainfall level is predicted for the next 100 years in Spain, less pronounced in coastal zones and islands (Castro et al. 2005). In keeping with Iberian *Calopteryx* ecological requirements and the distributions reported in this paper, the current climate change may severely affect their populations. Effects may be especially serious in the least thermal species, *C. virgo meridionalis. C. virgo* seems to be adapted to relatively cold waters, since it grows faster at low temperatures and has a higher standard metabolism than *C. splendens* (Schütte and Schrimpf 2002). Therefore, *Calopteryx virgo meridionalis* populations might be displaced to higher latitudes and/or higher altitudes. For instance, distributional range modification was clearly observed in Great Britain between 1960 and 1995: northwards expansion was higher in *C. virgo* (Hickling et al. 2005), since *C. splendens* prefers higher temperatures. Southern peninsular populations of *C. virgo meridionalis*, which are severely fragmented, are especially threatened by climate warming. A decrease in distributional range and possible local extinctions may be expected. These new vacant habitats (free of competitors and with new optimal conditions) might be occupied by *C. xanthostoma* or *C. haemorrhoidalis* (more thermal species). *C. xanthostoma* occurs in medium river courses, so its expansion is not as clear as that of *C. haemorrhoidalis.* A total reorganization of species distributions is likely. Intra- and interspecific interactions are especially marked in this family, so shifts in species distribution may involve profound changes in these interactions, affecting also interspecific dynamics. However, genetic studies need to be conducted to clarify the level of hybridization and genetic diversity in isolated populations, whose likelihood of survival might be compromised.

The use of bioclimatic belts to predict species distributions may be applied to other lotic species, especially endangered species. Although data for these species are usually scarce and disperse (except for some countries with traditional monitoring programs), this method may be applied to obtain preliminary results of species distributions. Specific variables should be considered to create accurate predictive models. However, not only physical variables may predict a species distribution, but also the association with other species, for which more data might be available. This association may therefore be used as a first step to assess the appropriate conservation status for little-known species. In addition, the obtained distributions and the association with bioclimatic belts may be used to study temporal series, consider past distributions and predict future changes in species distribution (especially outstanding within a global climate change context). Finally, a species distribution and its relation with other related species distributions must be considered in terms of evolutionary biology, considering its role as a cause of interpopulation variability and ultimately in speciation.
